# Augmenting antibody response to EGF-depleting immunotherapy: Findings from a phase I trial of CIMAvax-EGF in combination with nivolumab in advanced stage NSCLC

**DOI:** 10.3389/fonc.2022.958043

**Published:** 2022-08-03

**Authors:** Rachel Evans, Kelvin Lee, Paul K. Wallace, Mary Reid, Jason Muhitch, Askia Dozier, Circe Mesa, Patricia L. Luaces, Orestes Santos-Morales, Adrienne Groman, Carlos Cedeno, Aileen Cinquino, Daniel T. Fisher, Igor Puzanov, Mateusz Opyrchal, Christos Fountzilas, Tong Dai, Marc Ernstoff, Kristopher Attwood, Alan Hutson, Candace Johnson, Zaima Mazorra, Danay Saavedra, Kalet Leon, Agustin Lage, Tania Crombet, Grace K. Dy

**Affiliations:** ^1^ Roswell Park Comprehensive Cancer Center, Buffalo, NY, United States; ^2^ Department of Medicine Indiana University Melvin and Bren Simon Comprehensive Cancer Center, Indianapolis, IN, United States; ^3^ Centro de Immunologia Molecular, La Habana, Cuba; ^4^ National Cancer Institute (NCI) Division of Cancer Treatment and Diagnosis, Bethesda, MD, United States

**Keywords:** immunotherapy, lung cancer, non-small cell lung cancer, immune checkpoint inhibitor, vaccine

## Abstract

**Background:**

CIMAvax-EGF is an epidermal growth factor (EGF)-depleting immunotherapy which has shown survival benefit as a switch maintenance treatment after platinum-based chemotherapy in advanced non-small cell lung cancer (NSCLC). The primary objective of this trial is to establish the safety and recommended phase II dose (RP2D) of CIMAvax-EGF in combination with nivolumab as second-line therapy for NSCLC.

**Methods:**

Patients with immune checkpoint inhibitor-naive metastatic NSCLC were enrolled using a “3+3” dose-escalation design. Toxicities were graded according to CTCAE V4.03. Thirteen patients (one unevaluable), the majority with PD-L1 0%, were enrolled into two dose levels of CIMAvax-EGF.

**Findings:**

The combination was determined to be safe and tolerable. The recommended phase 2 dose of CIMAvax-EGF was 2.4 mg. Humoral response to CIMAvax-EGF was achieved earlier and in a greater number of patients with the combination compared to historical control. Four out of 12 evaluable patients had an objective response.

## Introduction

Cancer has been a large contributor to morbidity and mortality rates with lung cancer being the most frequent cancer worldwide (1). Non-small cell lung cancer (NSCLC) makes up around 80% of lung cancer diagnoses (2), with 57% of the cases diagnosed at an advanced stage (3). Cytotoxic chemotherapy is the foundation for majority of the treatment options for advanced NSCLC. More recently, advances in immunology and molecular biology have led to the development of targeted therapy and immune-activating therapies in NSCLC (3–8). Despite improved clinical outcomes with newer treatments, long-term 5-year survival rates remain dismal for NSCLC patients, hence the ongoing need for better therapeutics (4). CIMAvax-EGF is a recombinant anti-human epidermal growth factor (EGF)-depleting immunotherapy conjugated to the recombinant p64K (rP64K) protein derived from *Neisseria meningitidis* bacteria (5). The adjuvant (Montanide ISA 51 VG) is added to prepare the injection for administration and acts to enhance the immune response (6). CIMAvax-EGF induces the development of anti-EGF antibodies leading to depletion of circulating EGF with a very good safety profile (7–11). In a randomized study, CIMAvax-EGF as a switch maintenance treatment compared to best supportive care alone after first-line platinum-based chemotherapy for metastatic NSCLC demonstrated superior overall survival, particularly in patients with high baseline circulating serum EGF levels (11). Nivolumab is an IgG4 fully human monoclonal antibody that targets the programmed cell death protein 1 pathway (PD-1/PD-L1) (12). It is the first anti-PD-1 agent to be approved in NSCLC as second-line therapy and has also been approved for other indications such as melanoma, renal cell carcinoma, and squamous cell carcinoma of the head and neck (13). Previous studies (CheckMate 017 and CheckMate 057) demonstrated that nivolumab provides superior long-term clinical benefit in patients with metastatic NSCLC, favorable side effect profile, and sustained tolerability compared to docetaxel (14).

The impetus behind the development of CIMAvax-EGF with nivolumab is to investigate the potential synergy between these therapies. B lymphocytes are most important in an immune response for the production of antibodies against a specific antigen (15, 16). Nivolumab prevents binding of PD-1 to ligands PD-L1/L2 and thereby removes PD-1 ligand-mediated inhibition of T-cell proliferation function. Whether this results in enhancement of humoral response from B-cell activity has not been well characterized. Switched memory B cells have shown to be enriched in tumors that have responded to immune checkpoint blockade (17). By combining a B-cell vaccine with an immune checkpoint inhibitor, two different cell types, B lymphocytes and T lymphocytes, are targeted to promote and activate anti-tumor immunity. This combination may lead to an enhanced and more enduring immune response. Conversely, EGFR signaling in several models results in tumor PD-L1 expression (18) such as *via* glycosylation changes which stabilize the protein. A negative effect of N-glycosylation on binding between therapeutic PD-L1 antibodies on N-glycosylated PD-L1 has also been recently observed (19).

We report the results of a phase I clinical trial investigating the combination of CIMAvax-EGF with nivolumab as second-line therapy in metastatic NSCLC. The primary objective of the phase I study is to determine the safety profile and to establish a recommended phase II dose of CIMAvax-EGF in combination with nivolumab, based on dose-limiting toxicities (DLTs) as assessed by the Common Terminology Criteria for Adverse Events (CTCAE) v4.03. The secondary objectives include correlative studies for immune and antibody response as well as tumor response.

## Methods

### Study design and participants

This was a single-institution, open-label, phase 1 study evaluating dose escalation of two dose levels of CIMAvax-EGF. Enrollment followed a traditional 3 + 3 dose escalation design to evaluate the two dose levels of CIMAvax-EGF starting at dose level 1 of 1.2 mg until the maximum tolerated dose was found. Patients who did not have a DLT and did not complete the DLT period were replaced. Initially, three patients were enrolled at dose level 1. If no DLTs were observed after the DLT period had ended, then three new patients were to be escalated to dose level 2. If ≥2 DLTs were observed, then three new patients were to be de-escalated to dose level −1. If 1 DLT was observed, then an additional three patients were to be enrolled at dose level 1. In those six patients at dose level 1, if ≤1 DLT was observed attributable to the combination or CIMAvax alone, we were to proceed to dose level 2; otherwise, we were to proceed to dose level −1.

To be eligible for the study, patients should be ≥18 years of age and had a pathologically confirmed diagnosis of NSCLC as defined by the American Joint Committee on Cancer staging system—TNM 7th edition, 2010 (stage IIIB or IV). Patients were eligible for treatment with nivolumab as standard of care and had disease progression during or after platinum-based chemotherapy. If EGFR or ALK genomic tumor aberrations were present, patients must have progression on FDA-approved targeted therapy for these aberrations prior to receiving nivolumab. Adequate bone marrow and organ function was required. Patients of childbearing potential agreed to use adequate contraceptive methods. Following a dose-limiting toxicity of grade 3 myocarditis found in dose level 1, the eligibility criteria were updated to require cardiac markers within normal limits for inclusion.

Patients with any recent major surgeries, anticancer chemotherapy, radiation, or investigational agents were excluded unless a protocol-defined washout period was met. Patients with treated brain metastases were eligible as long as there is no need for corticosteroids within 4 weeks prior to starting the study treatment. They also must not have received any previous immunotherapy and could not have known immunosuppressive disease, active infection, or other serious uncontrolled medical conditions. Excluded cardiac history included myocardial infarct and arterial or venous thromboembolic events within 6 months, unstable angina, New York Heart Association class III or IV disease, and documented congestive heart failure; history of cardiomyopathy and uncontrolled hypertension; history of myocarditis; and history of cardiac surgery or ventricular arrhythmias. Patients were also excluded if they have been diagnosed with other invasive cancers within 2 years with exceptions and were pregnant or nursing mothers.

All participants gave written informed consent according to federal and institutional guidelines.

### Treatment administration

CIMAvax-EGF was investigated in two dose levels (1.2 and 2.4 mg). During the loading phase, CIMAvax-EGF was administered intramuscularly every 2 weeks for the first 4 doses. It was then administered monthly during the maintenance phase following the loading phase to coincide with nivolumab dosing. Nivolumab was administered every 2 weeks intravenously at 240 mg sequentially after CIMAvax-EGF on the days that both drugs are scheduled to be given. Patients remained on treatment until disease progression, DLT or serious toxicity, or withdrawal of consent. Patients were observed 1 h after administration of the treatment for assessment of vital signs and injection sites.

### Outcomes and procedures

#### Safety

Toxicities were graded according to the National Cancer Institute Common Toxicity Criteria version 4.03. The DLT evaluation period is defined as the first 4 weeks of treatments after completion of two doses of both CIMAvax-EGF and nivolumab. DLTs were defined as the occurrence of grade 3 or 4 treatment-related toxicity or intolerable grade 2 toxicity despite optimal supportive care. All adverse events, serious adverse events, and DLTs were reported as part of the investigational new drug (IND) FDA requirements.

#### Baseline and follow-up studies

A complete evaluation including medical history, physical exam, hematology, chemistry, cardiac safety markers (troponin-I, BNP, CK-MB), correlative markers, echocardiogram, urinalysis, pregnancy test in women of childbearing potential, ECOG status, 12-lead electrocardiogram, disease assessment (CT scan of the chest, abdomen, and pelvis and MRI of the brain with contrast), and concomitant medications was conducted within 30 days prior to the first dose of the study drug. Evaluations performed during the loading phase every 2 weeks included correlative markers, concomitant medications, and adverse events. Evaluations performed monthly during the loading phase included physical exam, ECOG, hematology, and chemistry. After a DLT of grade 3 myocarditis occurred, the schedule was updated to include cardiac safety markers and ECGs to be done periodically. Correlative markers and disease assessment were performed at the end of the loading phase. Evaluations performed monthly during the maintenance phase included correlative markers, physical exam, concomitant medications, ECOG, hematology, and chemistry. Disease assessment was conducted every other month. irRECIST was used to assess and document tumor response.

Follow-up safety evaluations and end of treatment evaluations occurred 30 days ( ± 14 days) after the participants had their last CIMAvax administration. Patients were followed up for 120 days after the end of treatments for changes in concomitant medication and adverse events.

#### Serial blood samples and immune biomarkers

EGF analyte concentrations in serum were measured using the Quantikine Human EGF Kit (R&D Systems, Minneapolis, MN, USA) according to the manufacturer’s instructions. Briefly, the quantitative ELISA used plates precoated with an anti-EGF mAb. Calibration standards, controls, and patient samples were added to individual wells and incubated 2 h at room temperature. After washing, an HRP-conjugated polyclonal antibody specific for EGF was added to the wells and incubated for 2 h. After a final wash step, the enzymatic reaction was visualized with tetramethylbenzidine substrate solution, and the absorbance was measured at 450 nm. Results (pg/ml) were calculated using a four-parameter logistic curve fit in GraphPad Prism v8.

Antibody titers against human EGF were measured by ELISA as previously described (20, 21). Unless otherwise indicated, incubations were for 1 h at 37°C in a humidified container. Microtiter plates were coated with 50 ng/well of human EGF (Center of Molecular Immunology, Havana, Cuba), incubated, and subsequently blocked with 1% BSA. Serially diluted serum samples were added in triplicate to the coated microtiter plates along with background, positive, and negative controls. After incubation, the plates were washed and incubated with a goat anti-human Ig-alkaline phosphatase antibody (Millipore Sigma, St. Louis, MO, USA). The enzymatic reaction was visualized by incubation with *p*-nitrophenyl phosphate for 30 min at 37°C, stopped with 3 M of sodium hydroxide, and measured at 405 nm. Anti-EGF antibody titer was defined as the inverse of the highest serum dilution, yielding a final absorbance value higher than the blank absorbance plus 3 SD.

Peripheral blood specimens collected in sodium heparin were immunophenotyped by flow cytometry as previously described (22). Briefly, on receipt, samples were washed once with FCM buffer (containing 0.5% BSA, 0.1% Na azide, and 0.004% disodium EDTA in PBS pH 7.2), resuspended to their original volume, and incubated for 10 min with normal mouse IgG (10 μg/test) to block Fc receptors. Cells were then aliquoted into tubes (100 µl per tube) and incubated for 20 min with an eight-color combination of the following mAbs: CD3 PECy7, CD4 PECy7, CD11b FITC, CD19 PECy7, and CD33 PECy7 purchased from Beckman Coulter (Miami, FL, USA); CD3 BV510, CD4 APCH7, CD4 BV510, CD8 PcPCy5.5, CD14 APCH7, CD15 V450, CD16 PE, CD25 BB515, CD27 APC, CD28 PE, CD31 BB515, CD138 BV421, CD45RA APC, CD45RO APC, CD45RO BV510, CCR6 BV510, CCR7 BV421, CXCR10 BB515, HLADR PcPCy5.5, IgD PcPCy5.5, and IgM BB515 from BD Bioscience (San Jose, CA, USA); CD19 BV510, CD45 PcPCy5.5, and CCR4 BV421 from BioLegend (San Diego, CA, USA); and CD39 PE, CD319 PE, and HLADR APC from Thermo Fisher (Waltham, MA, USA). Red blood cells were lysed with BD FACSLyse, and the resulting cell pellet was washed once with FCM buffer before fixing in 0.5% methanol-free formaldehyde (Polysciences, Warrington, PA, USA). Cytofluorometric analysis was performed using a BD FACSCanto flow cytometer with DiVa software equipped and quality-controlled daily with the CS&T software. For each tube, we strove to collect 250,000 events, sample permitting. Data were analyzed with WinList (Verity Software House, Topsham, ME, USA) using sequential gates to eliminate doublets, debris, aggregates, and either lymphocytes or mononuclear cells (MDSC panel) that were defined using a combination of forward scatter versus side scatter and CD45 versus side scatter.

Cytokines and other biomarkers were measured using the Luminex multiplex bead array assays. Millipore and R&D multiplex bead array panels were processed, and the raw data acquired, according to the respective manufacturer’s recommendations for sample preparation, sample dilution, reagent and standard curve preparation, incubations, and instrument settings. Incubations with magnetic analyte capture beads were performed overnight at 4°C for Milliplex assays and for 2 h at room temperature for the R&D panels. Washing steps were performed manually with a handheld magnetic 96-well plate washer according to instructions in the respective kit protocols. Raw data were acquired with a Luminex 200 instrument running xPonent acquisition software (version 3.1). Performance verification was completed on the Luminex instrument the morning of every assay read. Raw data were analyzed with Upstate BeadView software using the best fit curve-fitting equation for each analyte to calculate analyte concentrations from median fluorescent intensity (MFI) values in the test samples.

### Statistical methods

Patient characteristics and adverse events were summarized in the overall sample and by dose level using the mean, median, and range for continuous variables and using frequencies and relative frequencies for categorical variables. Overall and progression-free survival rates were summarized in the overall sample using the standard Kaplan–Meier methods. The association between the baseline biomarker values and the response and the survival outcomes were evaluated using logistic and Cox regression models, respectively. The models were fit using Firth’s method, and odds or hazard ratios, with 95% confidence intervals, were obtained from the model estimates. Analyses were performed in SAS v9.4 (Cary, NC, USA) at a significance level of 0.05.

### Data availability

Raw data for this study were generated at Roswell Park Comprehensive Cancer Center. Derived data supporting the findings of this study are available from the corresponding author upon request.

## Results

### Demographics

A total of 13 patients were enrolled in the phase 1 portion across two dose levels between 2016 and 2018. One patient (patient #12) was replaced in dose level 2 due to the requirement for steroids to manage symptomatic brain metastasis within the first week of the first dose. Descriptive baseline statistics are represented in **Table 1**.

**Table 1:** Descriptive baseline statistics by dose level.

		DOSE LEVEL 1	DOSE LEVEL 2	OVERALL
OVERALL COUNT	N	6	7	13
AGE	MEAN	59.1	62.6	61
	MEDIAN (RANGE)	63 (45-68)	63 (53-72)	63 (45-72)
SEX	MALE	1 (17%)	3 (43%)	4 (31%)
	FEMALE	5 (83%)	4 (57%)	9 (69%)
HISTOLOGY	SQUAMOUS	2 (33%)	0 (0%)	2 (15%)
	ADENOCARCINOMA	4 (47%)	6 (86%)	10 (77%)
	LARGE CELL	0 (0%)	1 (14%)	1 (8%)
SMOKING	FORMER	5 (83%)	6 (86%)	11 (85%)
	CURRENT	1 (17%)	1 (14%)	2 (15%)
	NEVER	0 (0%)	0 (0%)	0 (0%)
STAGE	IIIB	0 (0%)	0 (0%)	0 (0%)
	IV	6 (100%)	7 (100%)	13 (100%)
ECOG SCORE AT BASELINE	0	3 (50%)	3 (43%)	6 (46%)
	1	3 (50%)	4 (57%)	7 (54%)
BRAIN METS	PRESENT	3 (50%)	2 (29%)	5 (38%)
	ABSENT	3 (50%)	5 (71%)	8 (62%)
PRIOR RADIATION	THORAX/BONE	1 (17%)	3 (43%)	4 (31%)
EGFR	WILDTYPE	5 (83%)	7 (100%)	12 (92%)
	MUTANT	0 (0%)	0 (0%)	0 (0%)
	UNKNOWN*	1 (17%)	0 (0%)	1 (8%)
ALK	WILDTYPE	5 (83%)	7 (100%)	12 (92%)
	MUTANT	0 (0%)	0 (0%)	0 (0%)
	UNKNOWN*	1 (17%)	0 (0%)	1 (8%)
KRAS	WILDTYPE	3 (83%)	5 (71%)	8 (62%)
	MUTANT	1 (17%)	2 (29%)	3 (23%)
	UNKNOWN*	2 (33%)	0 (0%)	2 (15%)
PD-L1 STATUS AT BASELINE	>50%	0 (0%)	1 (14%)	1 (8%)
	>1 <25%	1 (17%)	1 (14%)	2 (15%)
	<1%	2 (33%)	5 (72%)	7 (54%)
	UNKNOWN	3 (50%)	0 (0%)	3 (23%)

*Unknown EGFR/ALK/ROS1/KRAS/PD-L1 status was in a patient with squamous histology. One patient with EGFR/ALK/ROS1 wildtype PD-L1 20% adenocarcinoma had insufficient tissue specimen for extended molecular testing.

### Safety and recommended phase II dose

There were no grade 4 or 5 toxicities that were attributed to either treatment. A summary of treatment-related toxicities can be found in **Table 2**. The first patient enrolled incurred an event of grade 3 myocarditis (LVEF 25%–30%) on cycle 1 day 8 and was determined to be related to nivolumab and unrelated to CIMAvax-EGF. The patient, thus, discontinued the protocol treatment after one dose. This was considered a DLT in dose level 1.

**Table 2:** Treatment-related toxicities in phase 1.

Adverse event	Grade 1	Grade 2	Grade 3	Any grade (*n* = 13)
injection site pain	5 (38.5%)	0 (0.0%)	0 (0.0%)	5 (38.5%)
Chills	3 (23.1%)	1 (7.7%)	0 (0.0%)	4 (30.8%)
Fatigue	3 (23.1%)	1 (7.7%)	0 (0.0%)	4 (30.8%)
Nausea	2 (15.4%)	1 (7.7%)	0 (0.0%)	3 (23.1%)
Pyrexia	2 (15.4%)	0 (0.0%)	0 (0.0%)	2 (15.4%)
decreased appetite	1 (7.7%)	1 (7.7%)	0 (0.0%)	2 (15.4%)
Vomiting	1 (7.7%)	1 (7.7%)	0 (0.0%)	2 (15.4%)
Diarrhea	1 (7.7%)	0 (0.0%)	0 (0.0%)	1 (7.7%)
Colitis	0 (0.0%)	1 (7.7%)	0 (0.0%)	1 (7.7%)
Pruritus	2 (15.4%)	0 (0.0%)	0 (0.0%)	2 (15.4%)
Rash	1 (7.7%)	0 (0.0%)	0 (0.0%)	1 (7.7%)
Arthralgia	0 (0.0%)	1 (7.7%)	0 (0.0%)	1 (7.7%)
flu-like illness	1 (7.7%)	0 (0.0%)	0 (0.0%)	1 (7.7%)
Hyperthyroidism	1 (7.7%)	0 (0.0%)	0 (0.0%)	1 (7.7%)
Pneumonitis	0 (0.0%)	1 (7.7%)	0 (0.0%)	1 (7.7%)
Myocarditis	0 (0.0%)	0 (0.0%)	1 (7.7%)	1 (7.7%)

Regarding this DLT, this patient had no change in serum EGF and anti-EGF antibody titers on day 15 compared to baseline. On day 12, a cardiac MRI showed global myocardial edema and the LVEF improved to 60%–65% with steroids. On day 29, the patient had a cerebral hemorrhage due to brain metastasis which led to severe hemiparesis. The family of this patient decided on comfort measures due to the poor prognosis. Autopsy was performed after the patient expired which revealed metastasis to both cardiac ventricles with no evidence of myocarditis (likely resolved with steroids and ATG administered during the hospitalization). In retrospect, the inflammatory response was most likely an on-target effect. This DLT led to the addition of cardiac markers and ECGs to be followed more frequently throughout the trial for subsequent patients. There were no additional DLTs nor other cardiac events observed in the remaining patients. In terms of other grade 2 or higher immune-related adverse events, one patient with squamous NSCLC (patient #5) developed colitis approximately 7 months into protocol treatment. The patient had a partial response as best response to the combination treatment. Nivolumab was discontinued and the colitis eventually resolved with corticosteroids without recurrence. This patient also underwent consolidation thoracic radiation to the mediastinum and left lung mass during this period. There was a 3-month treatment-free interval before CIMAvax-EGF was resumed. One month after the patient restarted treatment with CIMAvax-EGF alone (coincidentally, this is approximately 2 months after the last fraction of radiation treatment), patient #5 developed grade 3 pneumonitis due to radiation requiring oxygen supplementation and steroids. Patient #5 continued maintenance dosing while on steroid taper and did not experience recurrent pneumonitis symptoms off steroids despite ongoing treatment with CIMAvax-EGF.

The recommended phase II dose was found to be nivolumab 240 mg every 2 weeks in combination with CIMAvax-EGF 2.4 mg for four doses followed every 28 days of injections which aligned with prior clinical trials that evaluated CIMAvax-EGF as monotherapy (7). Since the combination was found to be safe and tolerable, further investigation of the combination was warranted and the phase II portion was initiated.

### Patient outcomes

At least 54% of enrolled patients had a known PD-L1 tumor proportion score (TPS) of 0%. Two patients from each dose level had a partial response for a total of four (33%) patients out of 12 evaluable patients. For the seven patients who had a PD-L1 TPS of 0%, there was a 43% response rate with three of these patients achieving a partial response (PR). Another PR was achieved by a patient who had a PD-L1 TPS of 60%. A total of two patients achieved stable disease (SD) for a total of 50% disease control rate and 33% objective response rate in the entire evaluable patient population.

The current median overall survival (OS) for all patients was 13.5 (95% CI: 4.6–22.1) months. For patients (*n* = 10) who completed the loading phase of four doses of CIMAvax, the median OS was 18.3 (95% CI: 6.8–NR) months. Patients with EGFR/KRAS/ALK wild type had a median OS of 21.7 (95% CI: 1.8–NR) months, and patients with EGFR/ALK/STK11 wild type and KRAS mutation had a median OS of 12.1 (95% CI: 6.8–18.3) months. There are currently two patients who are long-term responders that are still receiving active treatment for over 3 years.

### EGF concentration and anti-EGF antibody titers

**Figure 1:**
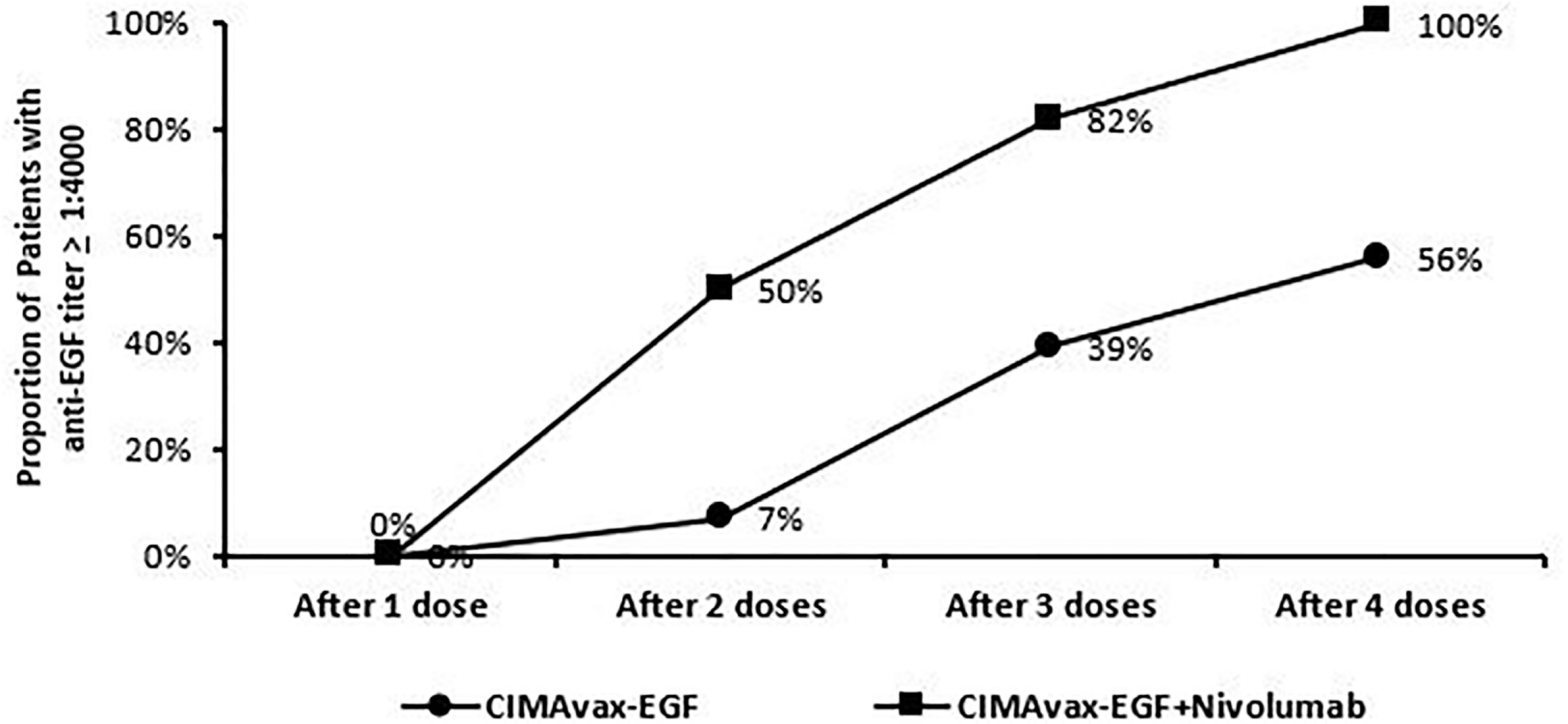
Trend of patients dosing and achieving anti-EGF titer ≥1:4,000. Good anti-EGF antibody response (≥1:4,000) elicited at earlier time points in more patients receiving CIMAvax-EGF in combination with nivolumab compared to historical controls.

**Figure 2:**
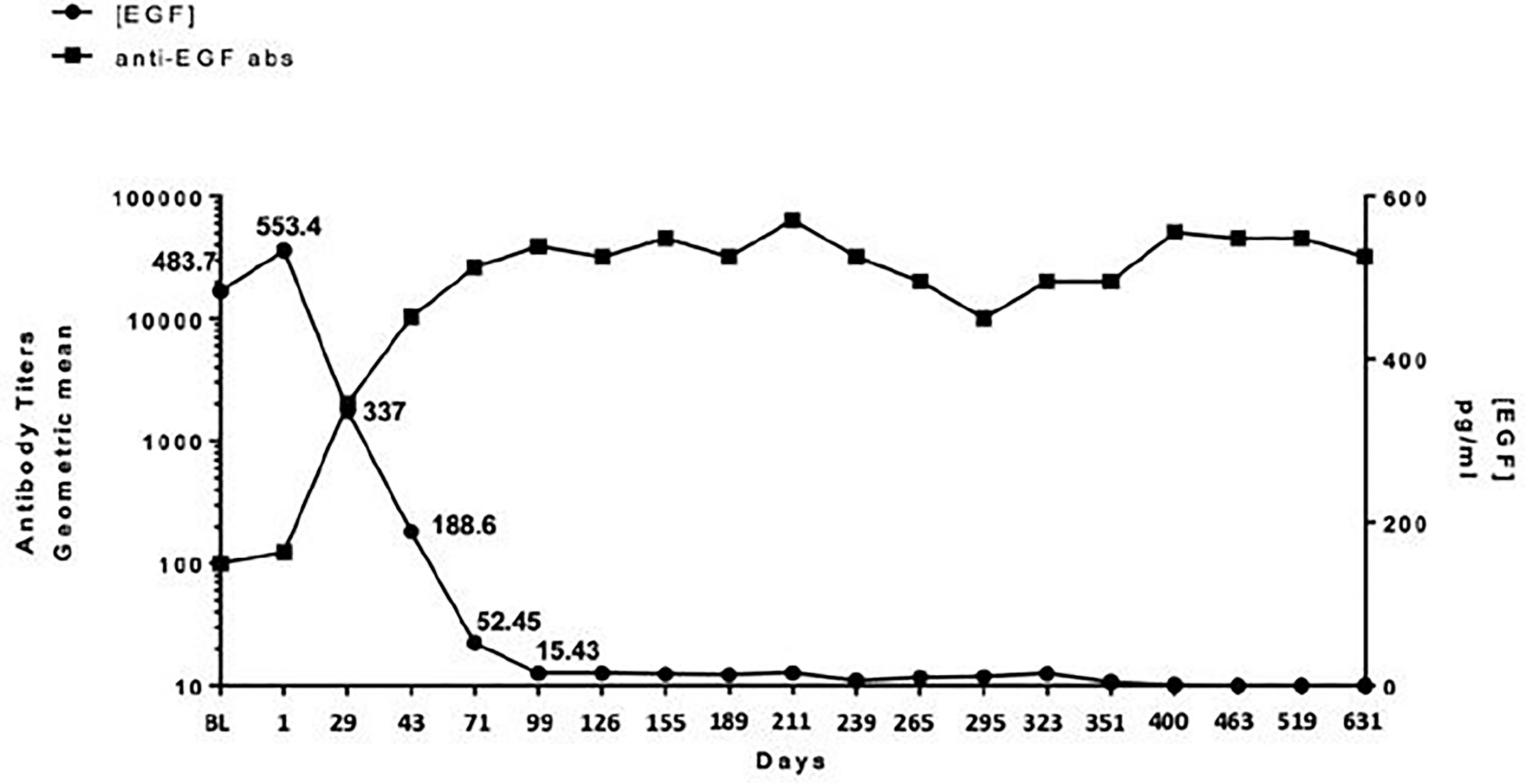
Relationship between anti-EGF antibody titers and serum EGF. There is a significant inverse correlation between circulating serum EGF and antibody titers in patients receiving CIMAvax-EGF in combination with nivolumab.

All enrolled patients had a baseline serum EGF level >250 pg/ml. The median level was 560 pg/ml with a range from 358 to 865 pg/ml. As defined in previous studies or trials with CIMAvax, patients who achieved anti-EGF titer levels ≥1:4,000 were identified as good antibody responders (GARs) (7). Excluding two patients who either received steroids during the loading phase and/or received less than three of the loading phase doses, 82% (9 out of 11) of the patients achieved GAR levels by day 43. All patients who received at least one maintenance dose (*n* = 8) reached antibody titers ≥1:4,000, of whom 25% (*n* = 2) achieved titers ≥1:64,000. Compared to a historical Cuban cohort receiving CIMA-vax-EGF alone, the combination of CIMAvax-EGF and nivolumab led to more patients achieving the GAR levels earlier. **Figure 1** compares the observed timeline of antibody titer response with historical controls (17). Based on a generalized linear model, there was a significant inverse correlation between anti-EGF antibody titers and serum EGF levels as expected, and this was previously demonstrated in the CIMAvax-EGF monotherapy trials (**Figure 2**). There was no correlation between the titer levels and the response to the combination of treatment (*p* = 0.21).

Durability and persistence of high titer levels in the maintenance phase without the need for continued dosing were serendipitously demonstrated in one of the responders who required overseas travel for personal reasons and skipped 6 weeks of protocol dosing (missed one maintenance of CIMAvax-EGF and three doses of nivolumab). Her titer levels were 1:128,000 prior to cycle 6 of therapy (missed cycle 7) and remained at the same level prior to cycle 8 dosing. The EGF levels remained suppressed as well: the pretreatment C1D1 level was 391 pg/ml, the pre-C6D1 level was 21 pg/ml, and the pre-C8D1 (despite missing the required doses over a 6-week period) was 24 pg/ml.

As expected, patients exposed to long-term high-dose corticosteroid therapy appear to be unable to mount or sustain a high antibody titer level. Patient #12 required steroids within the first week of protocol treatment due to worsening vision attributed to a treated brain metastasis located in the optic chiasm. Although this patient had to be replaced from a DLT evaluation perspective, he was permitted to continue on protocol treatment for compassionate reasons. Unlike other patients who demonstrated goal antibody titers after only three doses of CIMAvax-EGF, this patient’s antibody titer levels remained subtherapeutic (1:2,000) upon completion of the loading phase. The antibody response profile of patient #5, who required steroids for the management of colitis, demonstrated a sustained titer level of 1:8,000 about 2 weeks after the initial course of high-dose steroid therapy. The titer levels, however, declined to 1:4,000 after about 6 weeks of steroid treatment. This further declined to 1:2,000 a month later while the study treatment was on hold due to thoracic radiation. Although she regained the goal titer level after resuming treatment, the need for steroids again to manage radiation-related pneumonitis led to antibody levels being subtherapeutic 4 weeks later.

### Immune biomarkers

A comprehensive analysis of immune populations was performed by flow cytometric evaluation of peripheral blood samples obtained at baseline and serially after treatment. In the univariate analysis, there was a significant association with naive CD3^+^CD8^+^ T cells (CD45RA^+^CD28^+^CCR7^+^) and both overall survival (*p* = 0.046) and objective response (0.021), wherein higher levels at baseline were associated with objective response and improved overall survival. A similar trend was observed in the multivariate analysis but was no longer statistically significant (HR = 0.28, *p* = 0.99). In the multivariate models, a significant association was observed between IgM^−^IgD^−^CD127^−^ (CD27-Switched) and PFS, where increased expression was associated with poorer outcomes (*p* = 0.049). When comparing cell populations for titer response, significant associations were observed with CD11b^+^CD33^+^ HLADr(lo) CD14(lo) (*p* = 0.012), CD14^+^ monocytes (*p* = 0.033), classical monocytes (*p* = 0.033), and gate monocytes (*p* = 0.012). For all these markers, the responders tended to have a lower expression. Due to the small sample size relative to multicollinearity issues, multivariate models were not fit for the tumor response association.

**Figure 3:**
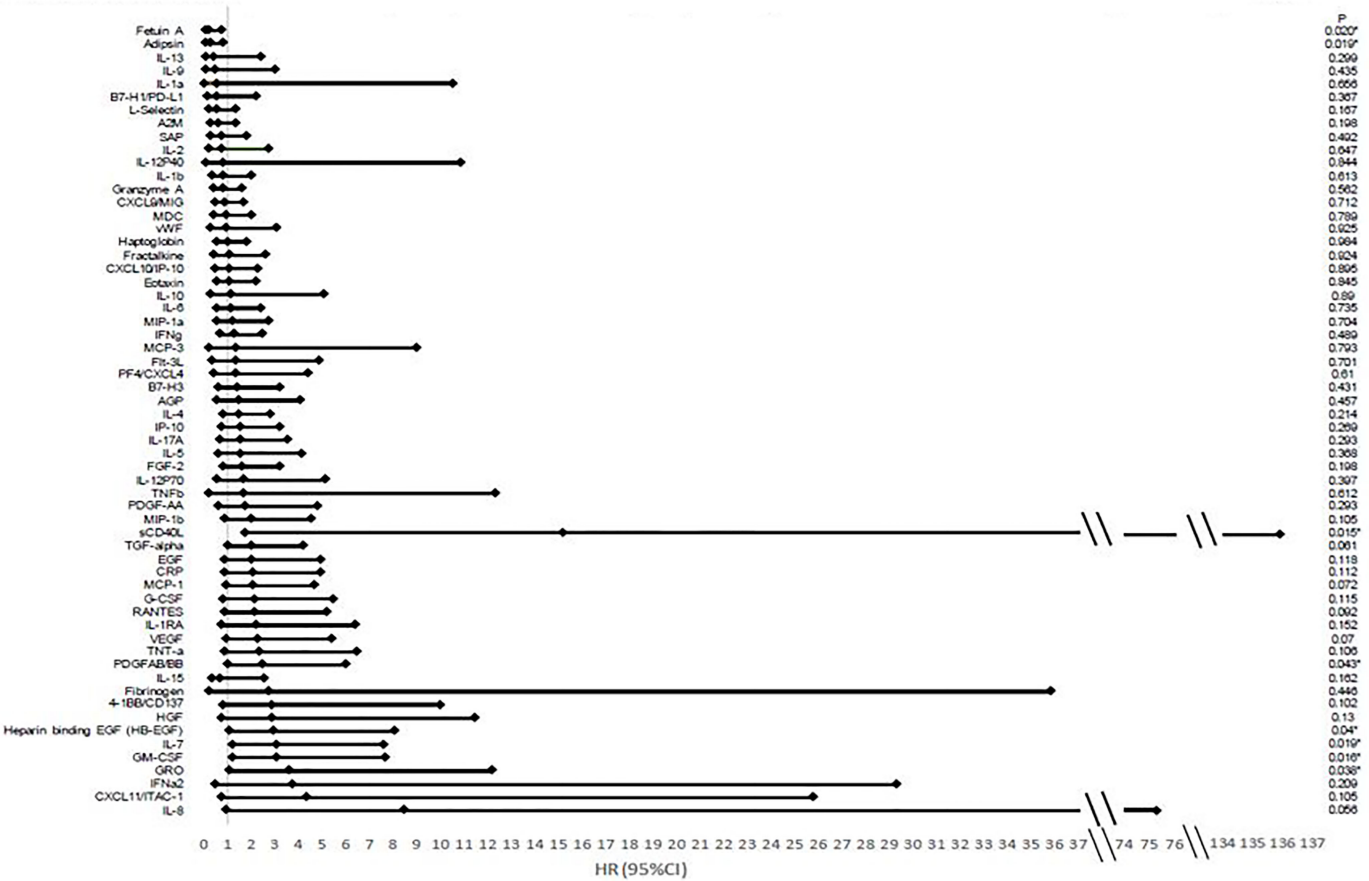
Hazard ratios and confidence intervals of cytokine association with overall survival. There is an association between many cytokines and overall survival based on analysis looking at cytokines as a continuous variable based on Luminex multiplex bead array assays. Hazard ratios (HRs) correspond to a standard deviation change in the cytokines.

Higher values of certain cytokine levels when analyzed as continuous variables were shown to be most associated with improved OS from a univariate analysis including adipsin and fetuin-A. Higher levels of GM-CSF, GRO, heparin-binding EGF (HB-EGF), IL-7, PDGFAB/BB, and sCD40L were associated with poorer outcomes (**Figure 3**).

## Discussion

Immune checkpoint inhibitor therapy has markedly changed the way advanced NSCLC is treated recently. Various factors influencing treatment outcomes with this class of immunotherapy have since been investigated (23, 24). PD-L1 is an imperfect predictive biomarker and there is concerted effort to incorporate other biomarkers [e.g., tumor mutation burden (TMB), tumor-infiltrating lymphocytes] to enhance patient selection, particularly for immune checkpoint inhibitor monotherapy (25–27). Although pre- and post-treatment tissues were not required nor analyzed for these patients during the dose-escalation portion, the ongoing phase II trial includes tumor tissue analysis of the tumor immune microenvironment, microbiome from stool samples, and circulating immune biomarkers both pre- and post-therapy for the combination of CIMAvax-EGF and immune checkpoint inhibitors.

This is the first published report on the use of CIMAvax-EGF combined with an anti-PD-1 from the phase III trial that was conducted in Cuba with CIMAvax-EGF monotherapy as switch maintenance therapy which showed that the majority of patients ultimately reached the GAR status defined as anti-EGF antibody titers equal or higher than 1:4,000 (7). Data from our clinical trial suggest enhancement of the antibody response, with the goal antibody titers reached in the majority (82%) of patients prior to the fourth dose of CIMAvax-EGF. This compares very favorably with the observed goal antibody response of 39% and 56% after three or four CIMAvax-EGF doses, respectively, in the Cuban phase III study (11). Although factors such as genetic, dietary, nutritional, and gut microbiome variability between the Cuban cohort and our NSCLC patients treated in continental USA may contribute to the observed difference in the timing of the humoral response seen, an additive effect induced by immune checkpoint blockade is mechanistically plausible based on preclinical models, and thus, this will equally if not more likely to explain this phenomenon (28, 29). Earlier achievement of GAR may also translate to better survival outcomes as seen in Checkmate 017 and CheckMate 057, and nivolumab has a five-fold increase in OS at 5 years compared to docetaxel (30). In our very small cohort of patients, it is encouraging to observe long-term responses in patients with PD-L1 0% tumors for which there was a 43% response rate in this subset of patients. This will need confirmation in a larger population. Our study was limited by not having information regarding additional biomarkers, such as TMB, as pretreatment biopsy was not required during dose escalation and the majority of patients had insufficient tissue for additional analysis. At the time the trial was designed, there were limited data regarding the use of immune checkpoint inhibitor therapy. Nevertheless, due to rapid changes in the standard of care in metastatic NSCLC wherein immune checkpoint inhibitor therapy is now incorporated in the frontline setting, the current phase II protocol will address this limitation and is amended to study pembrolizumab in combination with CIMAvax-EGF as first-line treatment in NSCLC patients with PD-L1 TPS ≥50%. A separate cohort investigating the combination with pembrolizumab as maintenance therapy in squamous NSCLC with PD-L1 <50% is also ongoing. Phase II will require pretesting biopsies to evaluate PD-L1 expression.

It is important to differentiate safety within the combination compared to nivolumab and CIMAvax-EGF alone. Historical data in patients treated with nivolumab alone have shown that most treatment-related adverse events occur within the first 3 months of treatment. The most common adverse events were fatigue, nausea, decreased appetite, and asthenia in the CheckMate 017 and CheckMate 057 studies (14, 30). Historical data in patients treated with CIMAvax-EGF showed that the most common adverse events seen were injection site reactions, fever, dyspnea, and vomiting (7). Our safety analysis was consistent with the historical data seen for each therapy alone and that the combination of these two therapies had no larger risk.

Although baseline serum EGF appeared to be a biomarker for selecting patients who can derive the most benefit from CIMAvax-EGF as switched maintenance treatment (11), there appeared to be no evident relationship between baseline nor temporal changes in serum EGF with treatment outcomes in our small cohort of patients progressing from first-line treatment. There was also no correlation between titer response and treatment response in this cohort. It is to be noted that since platelets sequester EGF which can be released into the serum during the coagulation process, stringent assay conditions must be observed to reduce variability related to the measurement bias itself. Our specimen procurement procedures and laboratory-developed assay underwent rigorous validation prior to the initiation of the study. Of interest is the serendipitous demonstration of durability of titer response that may obviate the need for monthly CIMAvax-EGF dosing. Conversely, even though there are conflicting data on the effect of corticosteroid therapy on humoral response to vaccination (31–33), our preliminary data suggest that high-dose corticosteroids can potentially adversely affect the generation and/or maintenance of sufficient antibody response.

Prior studies have shown that immune cell populations and cytokines could be useful to predict the responses to certain checkpoint inhibitors (34, 35). A previous phase II trial with nivolumab in melanoma cases has shown that IFN‐γ, IL‐6, and IL‐10 levels in the responders were significantly increased when compared to non-responders (*p* < 0.0001, *p* = 0.0007, and *p* < 0.0001, respectively). Our analysis showed that baseline levels of I CD8^+^ T cells were associated with treatment response as well as overall survival. Higher levels at baseline of this T-cell subset may increase the probability of activating and expanding the relevant tumor-specific effector T cells needed for tumor response upon reversal of immune checkpoint blockade (36). Lower levels of cytokines associated with inflammation lead to an increase in survival. Further research is needed as our sample size remains small. These associations may bring insight to further evaluate which population will benefit the most from this combination of therapies and could lead to predictive biomarkers that may be used when selecting therapy for these patients. Due to the small sample size, the early results from biomarker associations and clinical responses cannot be assumed to be a true association until it is further evaluated in future trials with larger sample sizes.

In conclusion, the combination of CIMAvax-EGF and nivolumab can be safely administered together at usual doses, with proof-of-concept demonstration of enhanced humoral response to a B-cell vaccine by inhibition of immune checkpoint *via* PD-1 blockade. A phase II trial is currently ongoing to investigate anti-PD-1 in combination with CIMAvax-EGF in a larger cohort of patients, including assessment of biomarkers in pre- and post-treatment tumor tissues.

## Data availability statement

The raw data supporting the conclusions of this article will be made available by the authors, without undue reservation.

## Ethics statement

The studies involving human participants were reviewed and approved by Roswell Park Institutional Review Board. The patients/participants provided their written informed consent to participate in this study.

## Author contributions

KeL, PW, MR, CM, OS-M, KaL, TC, and GD contributed to the conception and design of the study. RE, AD, IP, MO, CF, TD, ME, GD, CJ, and AL contributed to the implementation of the trial and collection of the patient data. PW, JM, CM, CC, AC, and DF contributed to the analysis of the samples. CM, PL, OS-M, AG, KA, AH, ZM, DS, and KaL contributed to the statistical analysis and analysis of patient data. RE wrote the first draft of the manuscript. All authors contributed to manuscript revision and read and approved the submitted version.

## Funding

This work was supported by a Developmental Funds award from the Roswell Park Alliance Foundation.

## Acknowledgments

We would like to thank Doug Plessinger, Thomas Schwaab, and Michael Sexton for their work in establishing the legal and drug supply infrastructure to enable drug sourcing amid an embargo.

## Conflict of interest

The authors declare that the research was conducted in the absence of any commercial or financial relationships that could be construed as a potential conflict of interest.

## Publisher’s note

All claims expressed in this article are solely those of the authors and do not necessarily represent those of their affiliated organizations, or those of the publisher, the editors and the reviewers. Any product that may be evaluated in this article, or claim that may be made by its manufacturer, is not guaranteed or endorsed by the publisher.

## References

[1] WHO. World cancer report. Geneva, Switzerland: World Health Organization (2014).

[2] Hughes BGM Chan BA. Targeted therapy for non-small cell lung cancer: current standards and the promise of the future. Trans Lung Cancer Res (2015) 4(1):36–54. doi: 10.3978/j.issn.2218-6751.2014.05.01 PMC4367711 25806345

[3] SEER cancer stat facts: Lung and bronchus cancer . Available at: https://seer.cancer.gov/statfacts/html/lungb.html.

[4] Van Damme V Govaerts E Nackaerts K Dooms C Wauters I Vansteenkiste J. Clinical factors predictive of long-term survival in advanced non-small cell lung cancer. Lung Cancer (2013) 79(1):73–6. doi: 10.1016/j.lungcan.2012.09.015 23083516

[5] Debra Evenson J. Cuba’s biotechnology revolution, Vol. 9. MEDICC Review and Online (2007). pp. 8–10, MEDICC Review, Fall. 10.37757/MR2007V9.N1.5 21487353

[6] Rodriguez G Albisa A Vinãa L Cuevas A Garcia B Garcia AT. Manufacturing process development for an epidermal growth factor-based cancer vaccine. BioPharm Int (2008) 2008(6).

[7] Rodriguez PC Popa X Martínez O Mendoza S Santiesteban E Crespo T. A phase III clinical trial of the epidermal growth factor vaccine CIMAvax-EGF as switch maintenance therapy in advanced non-small cell lung cancer patients. Clin Cancer Res (2016) 22(15):3782–90. doi: 10.1158/1078-0432.CCR-15-0855 26927662

[8] Pedro C Rodríguez M Gryssell Rodríguez MS González G Agustín Lage MD. Clinical development and perspectives of CIMAvax EGF, Cuban vaccine for non-small-cell lung cancer therapy. MEDICC Rev (2010) 12(1):17. doi: 10.37757/MR2010.V12.N1.4 20387330

[9] Gonzalez G Crombet T Torres F Catala M Alfonso L Osorio M. Epidermal growth factor-based cancer vaccine for non-small-cell lung cancer therapy. Ann Oncol (2003) 14(3):461–6. doi: 10.1093/annonc/mdg102 12598354

[10] Ramos TC Vinageras EN Ferrer MC Verdecia BG Rupalé IL Pérez LM. Treatment of NSCLC patients with an EGF-based cancer vaccine. Cancer Biol Ther (2006) 5(2):145. doi: 10.4161/cbt.5.2.2334 16357522

[11] Danay Saavedra EN Rodriguez C Viada C Mazorra Z Lage A Crombet T. CIMAvax-EGF: Toward long-term survival of advanced NSCLC. Semin Oncol (2018) 45:34–40. doi: 10.1053/j.seminoncol.2018.04.009 30318082

[12] Postow MA Callahan MK Wolchok JD. Immune checkpoint blockade in cancer therapy. J Clin Oncol (2015) 33(17):1974–82. doi: 10.1200/JCO.2014.59.4358 PMC4980573 25605845

[13] Xia L Liu Y Wang Y. PD-1/PD-L1 blockade therapy in advanced non-Small-Cell lung cancer: Current status and future directions. Oncologist (2019) 24(Special Issue):S31–41. doi: 10.1634/theoncologist.2019-IO-S1-s05 PMC6394772 30819829

[14] Horn L Spigel DR Vokes EE Holgado E Ready N Steins a. Nivolumab versus docetaxel in previously treated patients with advanced non–Small-Cell lung cancer: Two-year outcomes from two randomized, open-label, phase III trials (CheckMate 017 and CheckMate 057). J Clin Oncol (2017) 35(35):924–3933. doi: 10.1200/JCO.2017.74.3062 29023213 PMC6075826

[15] Clem AS. Fundamentals of vaccine immunology. J Glob Infect Dis (2011) 3(1):73–8. doi: 10.4103/0974-777X.77299 PMC3068582 21572612

[16] Sarkander J Hojyo S Tokoyoda K. Vaccination to gain humoral immune memory. Clin Transl Immunol (2016) 5(12):e120. doi: 10.1038/cti.2016.81 PMC5192068 28090322

[17] Helmink BA Reddy SM Gao J Zhang S Basar R Thakur R. B cells and tertiary lymphoid structures promote immunotherapy response. Nature (2020) 577(7791):549–55. doi: 10.1038/s41586-019-1922-8 PMC8762581 31942075

[18] Li CW Lim SO Xia W Lee H Li-Chuan C Chu-Wei K. Glycosylation and stabilization of programmed death ligand-1 suppresses T-cell activity. Nat Commun (2016) 7:12632. doi: 10.1038/ncomms12632 27572267 PMC5013604

[19] Lee HH Wang YN Xia W Chen CH Rau KM Ye L. Removal of n-linked glycosylation enhances PD-L1 detection and predicts anti-PD-1/PD-L1 therapeutic efficacy. Cancer Cell (2019) 36(2):168–178.e4. doi: 10.1016/j.ccell.2019.06.008 31327656 PMC6793936

[20] García B Neninger E de la Torre A Leonard I Martínez R Viada C. Effective inhibition of the epidermal growth factor/epidermal growth factor receptor binding by anti-epidermal growth factor antibodies is related to better survival in advanced non-small-cell lung cancer patients treated with the epidermal growth factor cancer vaccine. Clin Cancer Res (2008) 14(3):840–6. doi: 10.1158/1078-0432.CCR-07-1050 18245547

[21] Gonzalez G Crombet T Catala M Mirabal V Hernandez JG Gonzalez Y. A novel cancer vaccine composed of human-recombinant epidermal growth factor linked to a carrier protein: Report of a pilot clinical trial. Ann Oncol (1998) 9:431. doi: 10.1023/A:1008261031034 9636835

[22] Tario J Jr Wallace P. Reagents and cell staining for immunophenotyping by flow cytometry. In: McManus LM MR, editor. Pathobiology of human disease. San Diego: Elsevier (2014).

[23] Riaz N Havel JJ Makarov V Desrichard A Urba WJ Sims JS. Tumor and microenvironment evolution during immunotherapy with nivolumab. Cell (2017) 171:934–49. doi: 10.1016/j.cell.2017.09.028 PMC5685550 29033130

[24] Schalper KA Ricardo Diez-Valle M.E.R.-R. López-Janeiro A Porciuncula A Idoate MA Inogés S. Neoadjuvant nivolumab modifies the tumor immune microenvironment in resectable glioblastoma. Nat Med (2019) 25:470–6. doi: 10.1038/s41591-018-0339-5 30742120

[25] Alexander M GJ Cheng H. Tumor mutation burden in lung cancer: a new predictive biomarker for immunotherapy or too soon to tell. J Thorac Dis (2018) 10(Suppl 33):S3994–8. doi: 10.21037/jtd.2018.09.35 PMC6297473 30631537

[26] Greillier L TP Barlesi F. The clinical utility of tumor mutational burden in non-small cell lung cancer. Transl Lung Cancer Res (2018) 7(6):639–46. doi: 10.21037/tlcr.2018.10.08 PMC6249623 30505708

[27] Barlesi F Greillier L Monville F Foa C le Treut J Audigier-Valette C. LBA53 - precision immuno-oncology for advanced non-small cell lung cancer (NSCLC) patients (pts) treated with PD1/L1 immune checkpoint inhibitors (ICIs): A first analysis of the PIONeeR study. Ann Oncol (2020) 31:S1142–215. doi: 10.1016/j.annonc.2020.08.2286

[28] McKay JT Egan RP Yammani RD Chen L Shin T Yagita H. PD-1 suppresses protective immunity to streptococcus pneumoniae through a b cell–intrinsic mechanism. J Immunol (2015) 194(5):2289–99. doi: 10.4049/jimmunol.1401673 PMC4339454 25624454

[29] Bradley T Kuraoka M Yeh CH Tian M Huan C Derek WC. Immune checkpoint modulation enhances HIV-1 antibody induction. Nat Commun (2020) 11(1):948. doi: 10.1038/s41467-020-14670-w 32075963 PMC7031230

[30] Borghaei H Gettinger S Vokes EE Chow LQM Burgio MA de Castro Carpeno J. Five-year outcomes from the randomized, phase III trials CheckMate 017 and 057: Nivolumab versus docetaxel in previously treated non-Small-Cell lung cancer. J Clin Oncol (2021) 39(7):723–33. doi: 10.1200/JCO.20.01605 PMC8078445 33449799

[31] Hanania NA Sockrider M Castro M Holbrook JT Tonascia J Wise R. Immune response to influenza vaccination in children and adults with asthma: effect of corticosteroid therapy. J Allergy Clin Immunol (2004) 113(4):717–24. doi: 10.1016/j.jaci.2003.12.584 15100679

[32] Kubiet MA Gonzalez-Rothi RJ Cottey R Bender BS. Serum antibody response to influenza vaccine in pulmonary patients receiving corticosteroids. Chest (1996) 110(2):367–70. doi: 10.1378/chest.110.2.367 8697835

[33] Fairchok MP Trementozzi DP Carter PS Regnery HL Carter ER. Effect of prednisone on response to influenza virus vaccine in asthmatic children. Arch Pediatr Adolesc Med (1998) 152(12):1191–5. doi: 10.1001/archpedi.152.12.1191 9856428

[34] Yamazaki N Kiyohara Y Uhara H Iizuka H Uehara J Otsuka F. Cytokine biomarkers to predict antitumor responses to nivolumab suggested in a phase 2 study for advanced melanoma. Cancer Sci (2017) 108(5):1022–31. doi: 10.1111/cas.13226 PMC5448619 28266140

[35] Lena H Wolf J Cappuzzo F Zalcman G Baas JMP Farsaci B. Nivolumab in patients (pts) with advanced refractory squamous (SQ) non-small cell lung cancer (NSCLC): 2-year follow-up from CheckMate 063 and exploratory cytokine profiling analyses abstract 1370. J Thorac Oncol (2016) 11(4S):S113–42. doi: 10.1016/S1556-0864(16)30247-7

[36] Ahn E Araki K Hashimoto M Weiyan L James LR Cheung J. Role of PD-1 during effector CD8 T cell differentiation. Proc Natl Acad Sci U.S.A. (2018) 115(18):4749–54. doi: 10.1073/pnas.1718217115 PMC5939075 29654146

